# Optically Transparent Metamaterial Absorber Using Inkjet Printing Technology

**DOI:** 10.3390/ma12203406

**Published:** 2019-10-17

**Authors:** Heijun Jeong, Manos M. Tentzeris, Sungjoon Lim

**Affiliations:** 1 School of Electrical and Electronics Engineering, College of Engineering, Chung-Ang University, Seoul 06974, Korea; jhijun000015@gmail.com; 2 School of Electrical and Computer Engineering, College of Engineering, Georgia Institute of Technology, Atlanta, GA 30332, USA

**Keywords:** electromagnetic absorber, metamaterials, transparent absorber, inkjet printing

## Abstract

An optically transparent metamaterial absorber that can be obtained using inkjet printing technology is proposed. In order to make the metamaterial absorber optically transparent, an inkjet printer was used to fabricate a thin conductive loop pattern. The loop pattern had a width of 0.2 mm and was located on the top surface of the metamaterial absorber, and polyethylene terephthalate films were used for fabricating the substrate. An optically transparent conductive indium tin oxide film was introduced in the bottom ground plane. Therefore, the proposed metamaterial absorber was optically transparent. The metamaterial absorber was demonstrated by performing a full-wave electromagnetic simulation and measured in free space. In the simulation, the 90% absorption bandwidth ranged from 26.6 to 28.8 GHz, while the measured 90% absorption bandwidth was 26.8–28.2 GHz. Therefore, it is successfully demonstrated by electromagnetic simulation and measurement results.

## 1. Introduction

Metamaterials are artificial structures with infinitely arranged unit cells [1]. Since these types of structures can be used to control the permittivity and permeability of materials, they have many electromagnetic applications; for example, they are used in frequency-selective surfaces [2,3], super lenses [4,5], terahertz applications [6,7], artificial magnetic conductors [8,9], and high-impedance surfaces [10,11], physical sensors [12,13], chemical sensors [14,15], biosensors [16,17], invisible cloaking [18,19], imaging [20,21], antennas [22,23,24], and circuits [25,26,27].

Conventionally, high-loss materials have been used to fabricate electromagnetic wave absorbers. For example, a wedge-tapered absorber, which is based on ferrite or a composite material, can excellently absorb electromagnetic waves [28,29,30]. However, this absorber is bulky and costly. The Jaumann absorber was proposed in 1994 to overcome these drawbacks of the wedge-tapered absorber [31]. The Jaumann absorber is based on a resistive sheet and has a resonance structure; moreover, it has a small size and high absorptivity. However, the material size should be a quarter wavelength (λ/4), and, at low frequencies, it has a bulky size.

A metamaterial absorber [32,33,34] with a small thickness can have high absorptivity since wave absorption occurs through electromagnetic resonance. Furthermore, its fabrication is easy and involves low cost. Therefore, metamaterial absorbers have been researched in various electromagnetic fields such as stealth technology [35,36], electromagnetic interference/electromagnetic compatibility [37,38], or radio frequency (RF) sensor applications [39,40].

Recently, the development of transparent conductive materials has led to research on optically transparent electromagnetic devices [41,42,43]. This futuristic topic elicits interest in metamaterial absorber research because of its high performance in achieving transparency. In general, optically transparent metamaterial absorbers can be used for smart window applications. Because the main function of the window is optical transparency, the optically transparent metamaterial can absorb the electromagnetic wave at the specific frequency. Therefore, the proposed absorber can be used for smart windows of buildings or aircraft [44,45,46].

According to these demands, metamaterial absorbers have been researched actively for realizing optically transparent absorbers [46,47,48], and typically, indium tin oxide (ITO) films have been used for realizing such absorbers [44,49,50]. By using ITO films for preparing conductive patterns and the ground layer, a metamaterial absorber that is optically transparent and that has high absorptivity can be realized. However, the ITO film is costly and its fabrication process is complex. In particular, since the structure of a metamaterial absorber should have infinitely arranged unit cells, the practical use of ITO-film-based optically transparent metamaterial absorbers has limitations.

This paper proposes an optically transparent metamaterial absorber that can be fabricated using inkjet printing technology. In order to make the absorber optically transparent, thin conductive ring patterns were fabricated through inkjet printing technology, and the substrate used was a polyethylene terephthalate (PET) film having transparent properties. Inkjet printing [51,52,53] is an additive fabrication process that is cost effective and simple. Moreover, it can be used for printing on the any type of substrate. Thus, the proposed metamaterial absorber has the advantages of cost effectiveness and simplicity, unlike the fabrication process for ITO-film-based transparent metamaterial absorbers. The proposed optically transparent metamaterial absorber was successfully tested by performing a full-wave electromagnetic simulation and experimental measurements.

## 2. Numerical Simulations

**Figure 1:**
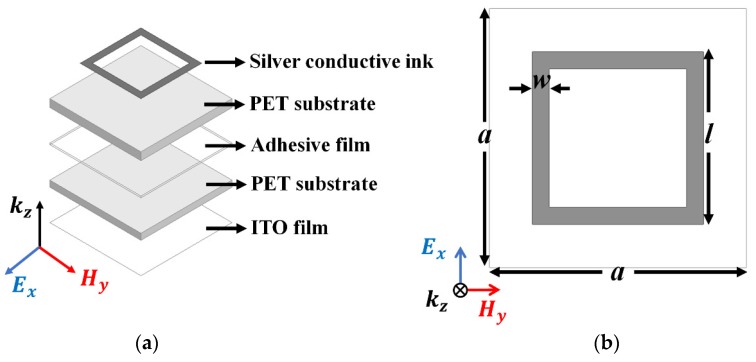

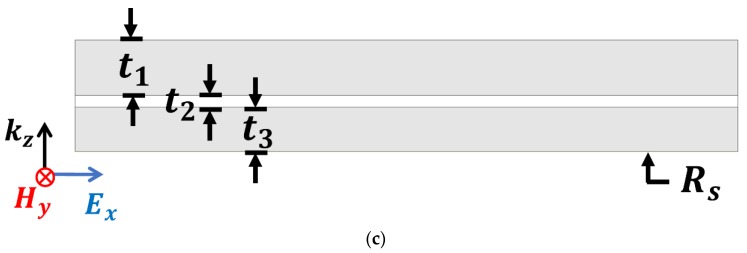
Geometry of the unit cell of the proposed absorber: (**a**) perspective, (**b**) top, and (**c**) side views of the unit cell.

The numerical simulation involving full-wave analysis by using the ANSYS High-Frequency Structure Simulator (HFSS, ANSYS, Washington, PA, USA) was used to design the proposed metamaterial absorber. Figure 1 shows the geometry of the unit cell of the proposed metamaterial absorber. To achieve the feature of optical transparency, apart from using two PET films (dielectric constant ε_r_ = 3 and loss tangent = 0.12) for the substrate and an ITO film for the bottom layer, as shown in Figure 1a, a thin square conductive loop was introduced with a width of 0.2 mm on the top layer of the PET substrate. The dimensions of the substrate and conductive pattern were *a* = 3 mm, *w* = 0.2 mm, and *l* = 2 mm, where *a* is the length of the substrate and *w* and *l* are the width and length of the conductive pattern, respectively. Adhesive tape of length 0.05 mm (t_2_) (ε_r_ = 3 and loss tangent = 0.05) was used to bind both PET substrates, as shown in Figure 1c. The thicknesses of the upper and lower PET substrates were t_1_ = 0.25 mm and t_3_ = 0.2 mm, respectively. The bottom layer was fully covered with a 5 Ω (R_s_) ITO conductive sheet to prevent wave transmission.

In order to achieve the best performance and quantify the sharpness, the parameter values were determined by conducting a parametric simulation study. The sharpness in the resonance can be defined by the following equation:

(1) $$\mathrm{Sharpness}\;\left(\text{Quality-factor}\right)=\frac{\mathrm{Resonance}\;\mathrm{frequency}}{3\mathrm{dB}\;\mathrm{bandwidtdh}}=\frac{f_{r}}{f_{2}-f_{1}},$$

where, *f_r_* is resonance frequency, *f*_1_ is lower frequency of 3 dB bandwidth, and *f*_2_ is higher frequency of 3 dB bandwidth, respectively.

**Figure 2:**
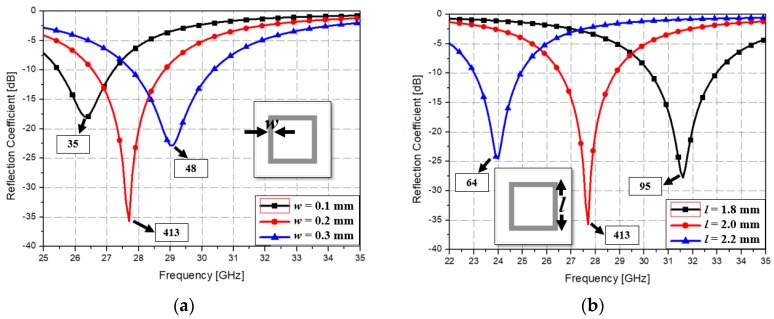

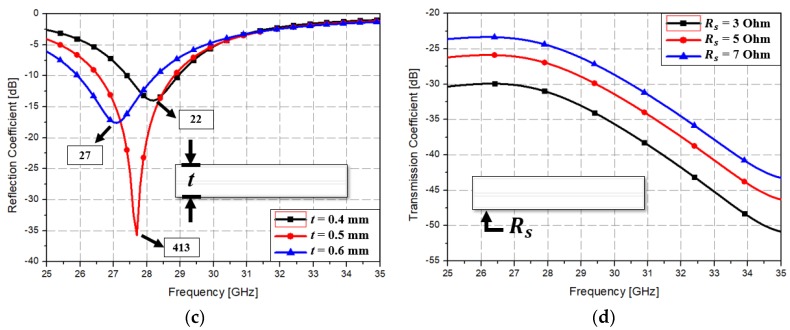
Plot of simulated S-parameters versus frequency for different values of parameters: (**a**) width of the square conductive loop, (**b**) length of the square conductive loop, (**c**) thickness of the unit cell, and (**d**) resistivity of the bottom ground plane.

Figure 2 shows the simulated S-parameters for different values of the parameters. When the conductive loop width was varied from 0.1 to 0.2, and 0.3 mm, the resonance frequency varied from 26.5 to 29 GHz and the sharpness varied from 35 to 413, and 48, respectively as shown in Figure 2a. the width was set at 0.2 mm on the basis of the proposed concept and the fabrication process capability. Next, when the length was varied from 1.8 to 2.0, and 2.2 mm, the resonance frequency varied from 31.5 to 24 GHz and the sharpness varied from 64 to 413, and 95, respectively as shown Figure 2b. The length was determined to be 2.0 mm to match the resonance frequency of 28 GHz. The thickness of the unit cell was varied to analyze the correlation between the thickness (t) of the substrate and the simulation result. When the thickness was varied from 0.4 to 0.5, and 0.6 mm, the resonance frequency slightly changed from 28 to 27 GHz and the sharpness varied from 27 to 413, and 95, respectively as shown in Figure 2c. Lastly, when the resistivity of the bottom ground plane was varied from 3 to 7 Ω, we observed that transmission coefficient varied from −31 to −27 dB and then to −24 dB, as shown in Figure 2d.

The absorptivity *A*(*ω*) can be defined as

(2) A(ω)=1−Γ(ω)−T(ω),

where Γ(*ω*) and *T*(*ω*) are the reflection coefficient and transmission coefficient, respectively. Since the proposed metamaterial absorber is fully covered with a conductive ground plane under the bottom layer, the transmission coefficient is zero. Therefore, the highest absorptivity performance of the metamaterial absorber can be achieved by minimizing the reflection coefficient, which can be defined as follows:

(3) $$\Gamma \left(\omega \right)=\frac{Z_{0}-Z_{M}}{Z_{0}+Z_{M}},$$

where Z_0_ is the free-space impedance (377 Ω) and Z*_M_* is the metamaterial absorber impedance. Thus, the highest absorptivity can be achieved when Z_0_ and Z*_M_* are equal, since the reflection coefficient is minimized.

**Figure 3:**
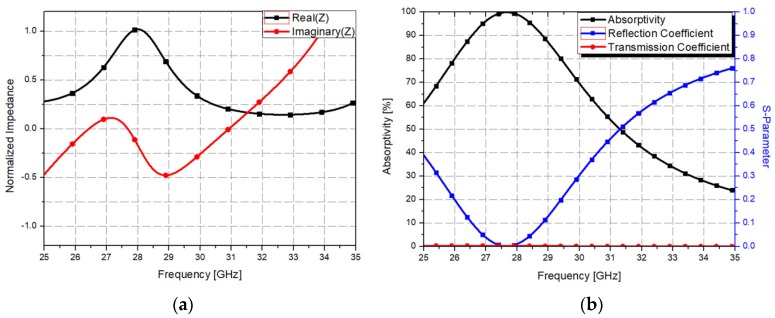
(**a**) Normalized impedance and (**b**) simulated S-parameters and absorptivity plotted against frequency.

In order to achieve the highest absorptivity performance, the normalized impedance and reflection coefficient was simulated; these parameters are shown in Figure 3. Figure 3a shows the normalized impedance. The proposed metamaterial absorber has 365.2 Ω of real impedance and −3.99 Ω of imaginary impedance at 27.7 GHz. Therefore, it can be observed that the normalized real impedance and normalized imaginary impedance are 0.95 and 0, respectively. The optimized impedance corresponds to the minimized reflection coefficient, as shown in Figure 3b. The proposed metamaterial absorber has a reflection coefficient of −35.1 dB at 27.7 GHz. Thus, we achieved 99.9% absorptivity at this frequency.

**Figure 4:**
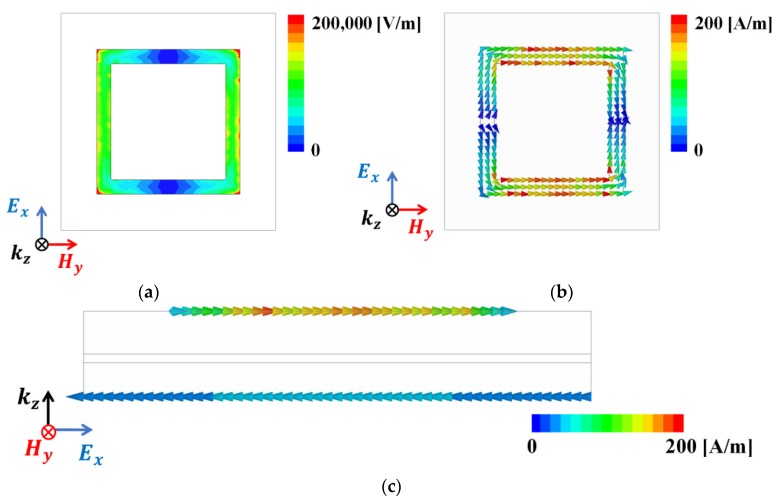
(**a**) Simulated electric field distribution: (**b**) top view and (**c**) side view of the vector current density at 27.7 GHz on the proposed metamaterial absorber.

Figure 4 shows a simulated electric field distribution and vector current density at 27.7 GHz. In Figure 4a, the electric field is strongly distributed at both edges of the square ring, implying that the width and length are the main factors determining the resonance frequency. This can be verified from Figure 2a,b. Similarly, the vector current shows strong flows at the top and bottom sides of the square ring (Figure 4b). Additionally, antiparallel flows were observed, which are part of a circulating loop as shown in Figure 4c.

**Figure 5:**
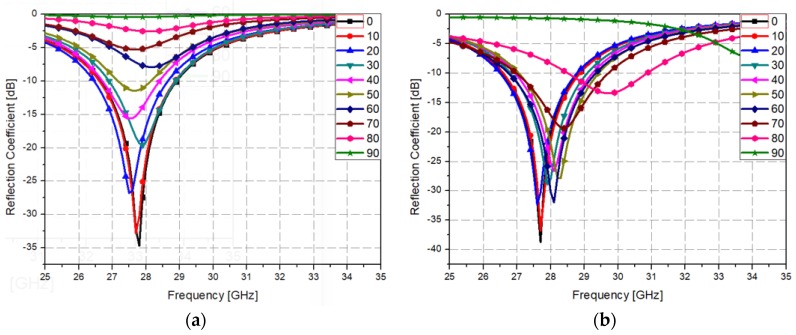
Simulated reflection coefficient of the proposed metamaterial absorber for different incident angles under (**a**) TE polarization and (**b**) TM polarization.

Figure 5 shows the simulated reflection coefficient of the proposed metamaterial absorber for different incident angles. The proposed metamaterial absorber has a 10 dB bandwidth from 26.6 to 28.8 GHz under normal incidence. Under oblique incidence, the 10 dB bandwidth is kept from 26.6 to 28.8 GHz at 10° in the transverse electric (TE) mode and 20° in the transverse magnetic (TM) mode. However, when the incident angle is varied from 0° to 90°, the 10 dB bandwidth is shifted from 26.4 to 28.5 GHz at 20° in the TE mode and from 26.8 to 29 GHz at 30° in the TM mode. Nevertheless, the angular stability of the proposed absorber is not competitive to other metamaterial absorbers with wider incidence angles [54,55,56] because we focused on the optical transparency in this work. An optical transparent metamaterial absorber with angular stability will be the next work.

## 3. Experimental Measurements

**Figure 6:**
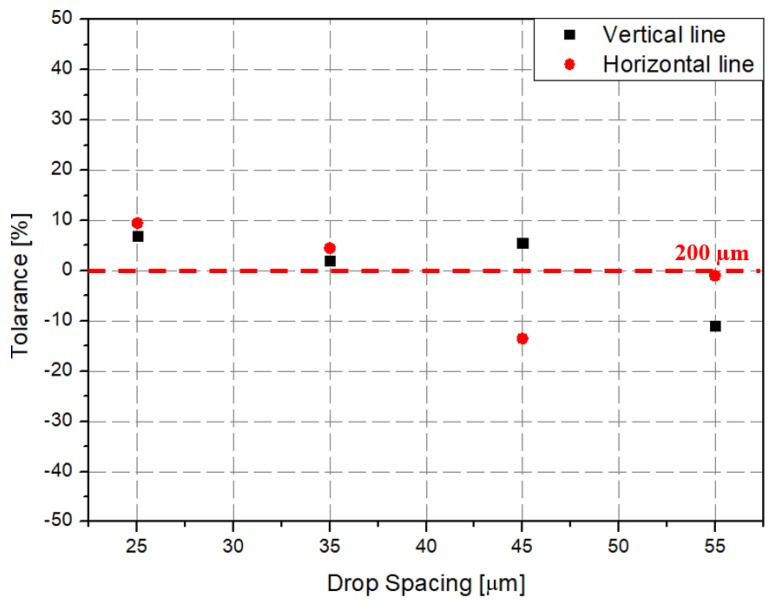
Comparison graph of vertical and horizontal line tolerance varying to the drop spacing.

To experimentally verify the simulation results, we fabricated a prototype of the metamaterial absorber. The conductive square ring was printed using a FUJIFILM Dimatix materials printer (DMP-2831, FUJIFILM, Minato, Tokyo, Japan) with a 1 pl cartridge (DMC-11601, FUJIFILM, Minato, Tokyo, Japan) and ANP silver nanoparticle ink (DGP 40LT-15C, ANP, Bugang-myeon, Sejong, Korea). To fabricate the 200 μm of line width, the fabricated line width was set as 150 μm because the printed line was a little bit spread. The vertical and horizontal lines are observed at different drop spacing as shown in Table 1. When the drop spacing is 15 μm, the vertical and horizontal lines are unstable because the ink spills out of the line. When the drop spacing is increased from 15 to 25 μm, it is observed from Table 1 that the vertical line is stable, but the horizontal line is not clear. When the drop spacing is increased from 25 to 35 μm, both vertical and horizontal lines are clearly printed. However, when the drop spacing is increased from 35 to 45, and 55 μm, the vertical and horizontal inks are leaking because the drop spacing is too far. Therefore, the drop spacing is set as 35 μm to realize the best line shape. In addition, when the drop spacing is set as 35 μm, the vertical and horizontal linewidth has only 2 and 4.5 percentages of tolerance, respectively, as shown in Figure 6. Finally, the cartridge head is set at 0° and the drop spacing is determined at 35 μm. In addition, three nozzles with 100 dpi resolution were used to print the designed pattern.

**Figure 7:**
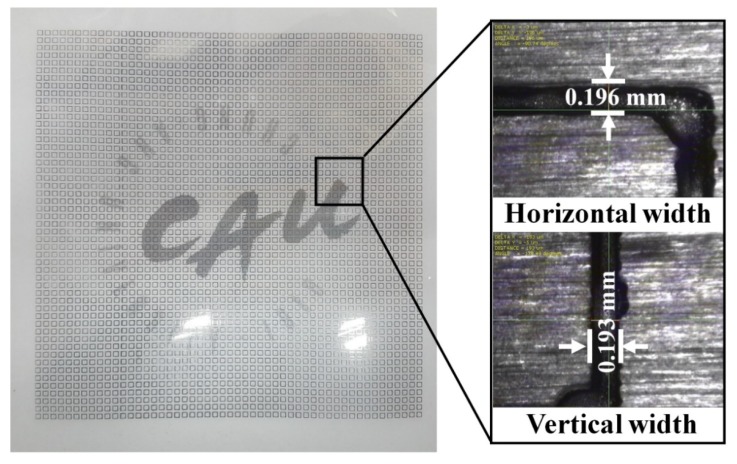
Fabricated prototype and magnified view of the printed surface.

Figure 7 shows the fabricated prototype and a magnified view of the printed surface. The prototype had dimensions of 198 mm × 198 mm and 67 × 67 unit cells. The horizontal and vertical widths of the printed surface were 0.196 and 0.193 mm, respectively, as shown in Figure 7, and they were very close to the value of 0.2 mm used in the simulation.

**Figure 8:**
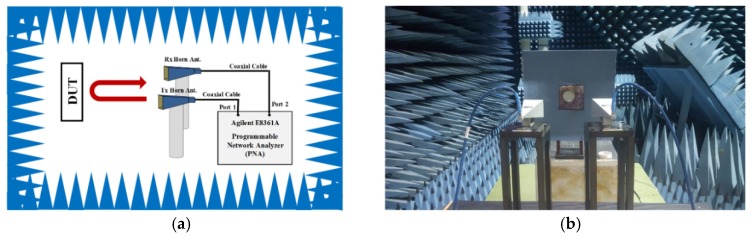
(**a**) Schematic and (**b**) a photograph of the measurement setup.

Figure 8 shows a schematic and a photograph of the measurement setup. For performing measurements of the prototype, the distance was set between the prototype sample and a horn antenna as 0.5 m for far-field conditions. To avoid unexpected reflected signals, the measurement was performed in an anechoic chamber and a Salisbury screen absorber was placed behind the sample. An Agilent E8361A programmable network analyzer (AGILENT, Santa Clara, CA, USA) and two horn antennas (frequency range: about 26.5–33 GHz) were used for the measurement, as shown in Figure 8a. The reflected signals were measured from the metamaterial plane and the reverse side of the ground plane. Next, the metamaterial plane and ground plane were compared to obtain the reflection coefficient, which was referenced to the reverse side of the ground plane.

**Figure 9:**
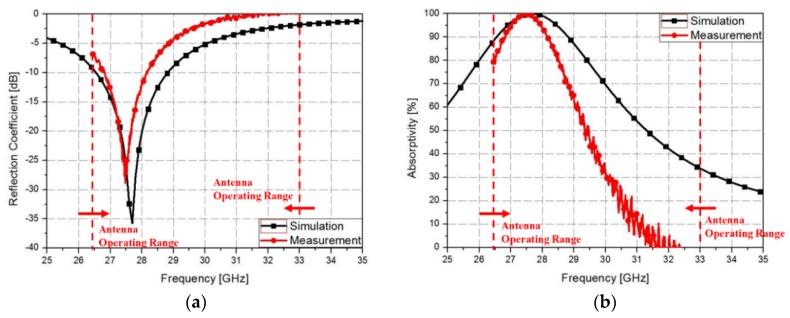
Comparison graph between simulation and measurement results: plots of (**a**) reflection coefficient and (**b**) absorptivity versus the frequency.

**Figure 10:**
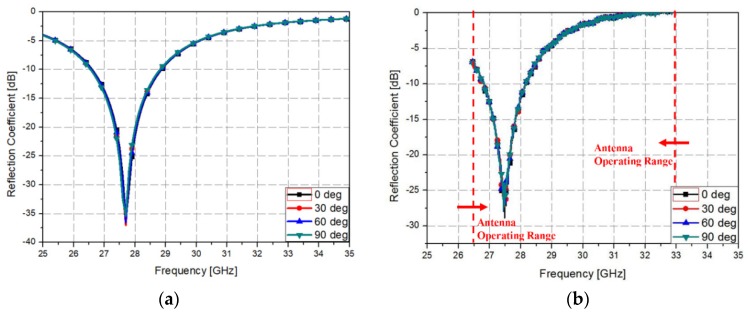
(**a**) Simulated and (**b**) measured reflection coefficients plotted against the frequency for different polarizations.

Finally, the measured reflection coefficients were obtained as shown in Figure 9a. The measured reflection coefficients had a bandwidth of −10 dB from 26.8 to 28.2 GHz, and the measured reflection coefficient at the resonance frequency of 27.5 GHz was −28.5 dB. Equation (2) was used for calculating the 90% absorption bandwidth, which ranged from 26.8 to 28.2 GHz as shown in Figure 9b. Since, the metamaterial absorbers have an infinite periodic structure, their absorptivity depends on the polarization angle of the incident electromagnetic wave. However, practical applications require an absorber whose performance can be kept constant even with varying polarization incidence. Therefore, metamaterial absorbers are required to have a polarization insensitive characteristic for the practical applications. Figure 10b shows the measurement of the prototype at various polarization angles to demonstrate its polarization insensitivity. As shown in Figure 10b, the prototype results hardly changed when the polarization angle was changed because the proposed metamaterial absorber was designed by symmetric structure.

To verify the excellence of the proposed work, the proposed optically transparent metamaterial absorber is compared with metamaterial absorbers proposed by other studies, and the comparison is shown in Table 2. As you can see in Table 2, the proposed metamaterial absorber has the advantage not only of being cost effective compared with using ITO sheet for conductor but also of advanced transparency compared with electro-textile or other metal mesh fabric methods. Hence, from the entire numerical simulation and experimental measurements, one can simply infer that the proposed metamaterial absorber has high absorptivity having polarization insensitivity.

## 4. Conclusions

This paper proposes an optically transparent metamaterial absorber fabricated using inkjet printing technology. To make the metamaterial absorber optically transparent, a thin conductive loop pattern was introduced on the top surface and PET films were used to fabricate the substrate. The thin conductive pattern was prepared using an inkjet printer, and its width was 0.2 mm. Thus, an optically transparent metamaterial absorber was realized with small width and optically transparent substrate. The metamaterial absorber was simulated using a full-wave electromagnetic simulator and measured with a free-space measurement setup. The numerical simulation indicated that the 90% absorption bandwidth of the metamaterial absorber ranged from 26.6 to 28.8 GHz, while experimental measurements yielded a range from 26.8 to 28.2 GHz. Furthermore, the proposed metamaterial absorber has a polarization insensitive characteristic. In conclusion, it is successfully demonstrated by the numerical simulation and measurement results.

## Figures and Tables

**Table 1:** Comparison of the fabricated vertical and horizontal line according to the different drop spacings.

Drop Spacing	Vertical Line	Horizontal Line
15 μm	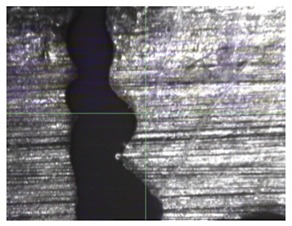 (unstable)	
25 μm	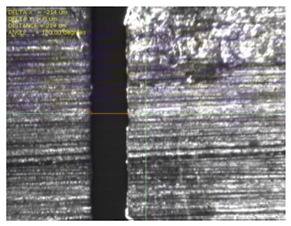	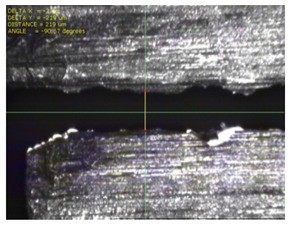
35 μm	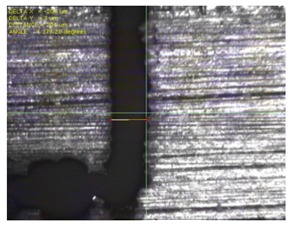	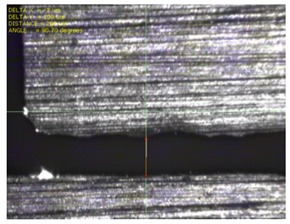
		
45 μm	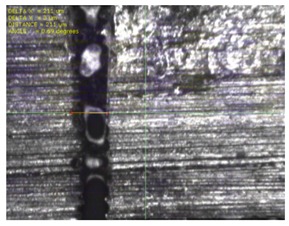	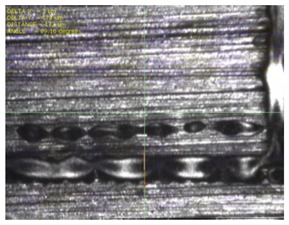
		
55 μm	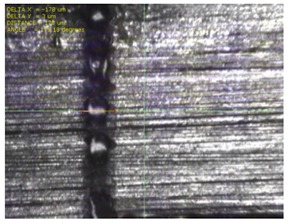	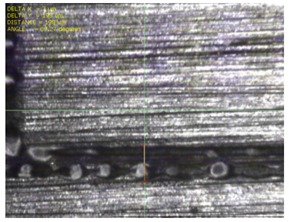

**Table 2:** Comparison of the proposed optically transparent metamaterial absorber with previously reported metamaterial absorbers.

Reference	Transparency Method	Frequency Range [GHz]	Cost	Transparency ^1^ (>90%)
[55]	Electro-textile on acryl substrate	27–35	Low	No
[56]	Metal mesh fabrics using screen printing	1.5–2.5	Low	No
[57]	Metal mesh fabrics on ceramic substrate	2.35–2.75	High	No
[54]	Metal mesh fabrics on glass sheet substrate	2.4, 3.7, 5.7	Low	No
[46]	ITO sheet	6.06–14.66	High	Yes
Present study	Metal mesh introduced using inkjet printing	26.8–28.2	Low	Yes

^1^ Transparency (%) = (1 − opaque area/total area) × 100; ITO: indium tin oxide.
